# Lost but Not Lost—Embolization of a Leadless Pacemaker to the Pulmonary Artery with Consecutive Endovascular Recovery

**DOI:** 10.3390/jcdd8040037

**Published:** 2021-04-07

**Authors:** Fabian Barbieri, Christof Kranewitter, Andreas Frech, Florian Hintringer, Markus Stühlinger

**Affiliations:** 1 Department of Internal Medicine III, Medical University of Innsbruck, 6020 Innsbruck, Austria; florian.hintringer@tirol-kliniken.at (F.H.); Markus.Stuehlinger@tirol-kliniken.at (M.S.); 2 Department of Radiology, Medical University of Innsbruck, 6020 Innsbruck, Austria; christof.kranewitter@tirol-kliniken.at; 3 Department of Vascular Surgery, Medical University of Innsbruck, 6020 Innsbruck, Austria; andreas.frech@tirol-kliniken.at

**Keywords:** transcatheter pacemaker system, device embolization, pulmonary artery, case report

## Abstract

Background: Leadless transcatheter pacemaker systems (TPS) have become a valuable alternative to transvenous pacemakers in selected indications. With the steadily increasing amount of TPS implantations performed worldwide, reports of periprocedural complications are likewise increasingly found in the literature but are still underreported. Case presentation: We report a case of a 75 year old male undergoing TPS implantation due to cardioinhibitory vasovagal syncope. The implantation was primarily uneventful; adequate pacing parameters and fixation of the device were achieved. Unfortunately, dislocation of the leadless pacemaker occurred at the end of the procedure and the device embolized into a primary side branch of the right pulmonary artery. Endovascular retrieval was performed by using a single snare technique without any further complications. Conclusions: Although challenging, endovascular recovery of embolized TPS from the pulmonary artery is feasible and may be successfully accomplished by experienced implanters.

## 1. Introduction

Cardiac pacemakers are the therapy of choice in patients suffering from symptomatic bradycardia [1]. Although a variety of refinements in implanting techniques and covering materials have been achieved over the last decades, lead-related complications such as fractures or insulation breaches, infections, and dislocations are common during long-term follow-up [2,3]. Transcatheter pacemaker systems (TPS), which are implanted directly into the ventricle, do not require the placement of transvenous leads and have been shown to overcome these complications [4]. While atrial pacing still remains unavailable in TPS, newer generation devices have also introduced an automated, enhanced accelerometer-based algorithm measuring atrial activity and therefore enabled atrioventricular synchronous pacing [5]. With the steadily increasing number of TPS implantations performed worldwide, reports of occurring complications are also increasingly found in the literature but are still underreported [6]. We herein present a case with an unintended dislocation of a TPS during implantation with consecutive embolization into the pulmonary artery and provide a review of currently available literature.

## 2. Case Presentation

A 75 year-old-male patient with a chronically implanted VVI pacemaker presented for routine assessment of his device in February 2018. The pacemaker had been implanted for recurrent cardioinhibitory vasovagal syncope in 1988, and the last generator change had been performed in 1996 (St. Jude Medical Regency SR 2404L; St. Jude Medical, MN, USA). The patient was asymptomatic and physically in good clinical condition; known co-morbidities included an atypical chronic lymphatic leukaemia with a stable disease since diagnosis in 2012 under a watch and wait strategy and an implantation of a hip prosthesis due to cox arthrosis. He did not report any regular medication. During pacemaker follow up, an intermittent increase in pacing threshold (up to 2.2 V at 1.0 ms) without any signs of insulation breach/fracture or loss of sensing was detected. Moreover, the battery had also reached the elective replacement indicator. Although the proportion of right ventricular pacing was less than 1%, the patient showed good clinical response to pacemaker therapy as there was no recurrence of syncope since beginning of treatment. Possible treatment strategies (generator change vs. TPS implantation) were discussed with the patient, and we decided to opt for a TPS according to current recommendations [7]. 

Verbal and written consent were obtained. Device implantation (Medtronic Micra^®^; Medtronic, Dublin, Ireland) was conducted in conscious sedation (propofol) and supportive local anaesthesia at the puncture site. The procedure itself was primarily uneventful achieving adequate pacing parameters (1.13 V at 0.24 ms, R-wave sensing 14 mV, impedance 720 Ohms). The pull and hold test verified movement of at least two tines confirming adequate fixation as seen in the supplementary materials (S1). Unfortunately, most probably due to friction whilst removing the tether, an accidental forward movement of the delivery tool led to dislocation of the leadless pacemaker, which further embolized into a primary side branch of the right pulmonary artery. An angiogram was conducted for proper visualization (S2), and strategies for endovascular retrieval were discussed in the catheter laboratory. The implanting team and the consulted interventional radiologist opted for a single-snare technique by primarily using a steerable sheath (Agilis, Abbott Laboratories, Abbott Park, IL, USA) inserted into the Micra^®^ delivery sheath to gain access to the right ventricular outflow tract. The steerable sheath was then exchanged for a 6 French 90 cm sheath (Brite Tip^®^, Cordis, Hialeah, FL, USA) and an Amplatz Goose Neck Microsnare Kit (ev3 Incorporation, Plymouth, MN, USA) to reach the leadless pacer. After access to the target pulmonary artery branch and multiple attempts, the device was finally caught at the proximal part with the snare catheter and retracted to the guiding sheath (S3). The sheaths, snare, and leadless pacer were subsequently pulled back to the site of puncture. Complete retrieval was achieved by venotomy and consecutive surgical repair as it was impossible to reinsert the device into the delivery sheath.

The patient remained haemodynamically stable throughout the procedure. Repetitive post-procedural vascular ultrasound yielded no therapeutically relevant abnormalities of the femoral veins. Echocardiographic examination showed only minimal pericardial effusion without any need of pericardiocentesis. At the follow-up one week after discharge, the decision for a generator change was made, which was conducted uneventfully two months later. Repetitive pacing threshold testing at every follow-up remained stable (1.0–1.5 at 0.4 ms) over the last two years. No further intervention was necessary.

## 3. Discussion

Leadless TPS are currently an alternative to transvenous pacemakers in selected indications [1,4]. Due to continuous improvements in these devices, it can be expected that current indications will be further expanded in the near future [5]. A low complication rate has been reported in large trials [4], however, there is high demand for related literature as they still might occur. Feasibility of endovascular recovery of a dislocated and/or embolized TPS has been previously demonstrated in case reports for all currently (Micra^®^, Wireless stimulation endocardial system (WiSE-CRT^®^); EBR Systems Inc., Sunnyvale, CA, USA) and formerly (Nanostim^®^, St. Jude Medical) available leadless pacing systems, but these procedures are described as challenging [8,9,10,11,12,13,14,15,16,17,18,19]. Dislocated right-sided devices (Micra^®^, Nanostim^®^) mostly embolize into the pulmonary arteries, affecting either side (left vs. right) with its corresponding branches equally often [8,9,10,11,12,13,14,15]. Two cases with floating devices have been described; one of them was the result of an attempted retrieval and subsequent dislocation to the right atrium [16], the other TPS was floating between right ventricular apex and tricuspid valve, resulting in repetitive non-sustained ventricular tachycardias [17]. Similar to our case, most devices were retrieved by using a single snare technique [8,9,10,11,12]. Alternatively, double snare techniques have been described to be successful as well [13,14,15,16,17,18], especially in cases of floating devices [16,17]. 

The complexity in performing the retrieval is mostly due to very distal positions of the TPS, absence of steerability of the sheaths in the right ventricular outflow tract and the pulmonary arteries, and difficulties to grasp the proximal knob of the device (e.g., upside-down position or floating devices). In these situations, it seems more favourable to start with a double snare technique from the beginning. Specifically, the tines should be caught with one snare and the retrieval feature grasped after fixation as a second step. By doing so, the tines of the TPS are then released before the device is reinserted into the introducer sheath [13,14,15,16,17,18]. Regarding the choice of catheters, most procedures have been conducted by either using a combination of a Micra^®^ introducer sheath with an angiographic catheter [9,15], a steerable sheath [16,18], or both [17]. Alternatively, two cases described the utilization of a multipurpose catheter, either in combination with an introducer sheath [14] or an introducer sheath and a steerable sheath [11]. In a single case, successful retrieval was possible by using a steerable sheath only [10]; two further cases did not specify the catheters used. An overview of potential techniques for early or immediate retrieval of non-dislocated TPS is described by Afzal et al. [20].

## 4. Conclusions

We found no trials about retrieval of embolized TPS in the literature. However, feasibility of endovascular retrieval in dislocated devices during or early after implantation has been described in case reports. There are currently no specialized extraction tools available, and techniques by using either a single or two snares have been used successfully. Especially after embolization into the pulmonary artery, the procedure remains challenging, but it may be achieved by experienced implanters.

## Data Availability

Data is contained within the article or supplementary material.

## Supplementary Materials

The following are available online, Video S1: Pull and hold test, Video S2: Angiography of the right pulmonary artery, Video S3: Successful retrieval of the device to the guiding sheath.
